# A new hypothesis may explain human parthenogenesis and ovarian teratoma: A review study

**DOI:** 10.18502/ijrm.v21i4.13267

**Published:** 2023-05-08

**Authors:** Abdelmonem Awad Hegazy, Aiman Ibraheem Al-Qtaitat, Raafat Awad Hegazy

**Affiliations:** ^1^Faculty of Dentistry, Zarqa University, Zarqa City, Jordan.; ^2^Faculty of Medicine, Zagazig University, Zagazig City, Egypt.; ^3^Faculty of Medicine, Mutah University, Alkarak, Jordan.

**Keywords:** Teratoma, Cloning, Parthenogenesis, Asexual reproduction.

## Abstract

Parthenogenesis (PG) is a rare phenomenon occurring in humans, and understanding this may help us develop an explanation for such occurrences. Moreover, it may help reveal the cause of idiopathic ovarian teratoma (OT). We aim to explain the occurrence of PG and OT in humans based on a new hypothesis. Previous literature has been searched through relevant scientific websites and international journals on the causes and mechanisms of PG and OT in humans. The previous literature on human PG was sparse and mostly contained case reports. It appears that human PG is not as rare as previously reported but may occur spontaneously, resulting in OT formation. The difference between PG and sexual reproduction is that PG has no embryonic diversity. The biopsied embryonic samples in the PG correspond exclusively to those of the maternal side. Spontaneous PG in humans often degrades or leads to formation of OT. The cause and mechanism of spontaneous PG remain unclear in the available literature. Here, we hypothesized that in some cases the secondary oocyte and first polar body enclosed in the zona pellucida may fuse together to form a single cell that restores the diploid number of chromosomes and initiates cell division to form PG. It may go unnoticed or be represented by the OT. Future studies are recommended to investigate this hypothesis.

## 1. Introduction

**Figure 1:**
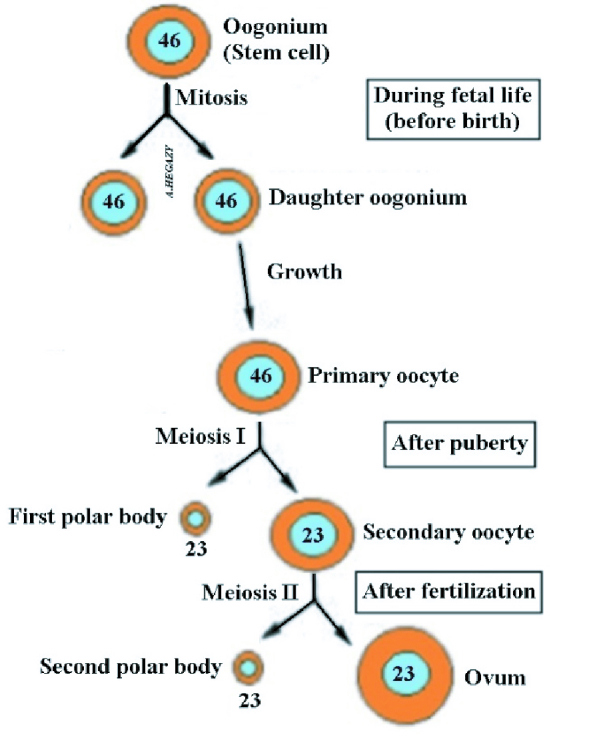
Diagrams showing oogenesis.

Parthenogenesis (PG) is an asexual reproduction in which a female can produce an embryo without fertilizing an egg with sperm. In Greek, it means the virgin creation. It occurs naturally in some jawed vertebrates such as the whiptail lizard, but in mammals, it is an unnatural event (1). However, PG may not be a rare event in humans as we thought, but it may occur spontaneously resulting in the formation of teratoma in the ovary (2). The secondary oocyte (SO), which is ovulated at each ovarian cycle, usually stops in metaphase II until it is fertilized by the sperm (Figure 1). Once the sperm enters the oocyte, the egg completes meiosis II to form the ovum, whose nucleus fuses with the sperm nucleus to form the zygote (3). This process is called sexual reproduction. However, under idiopathic abnormal circumstances, a spontaneous exit of the oocyte from metaphase-II could occur without apparent stimulation. This is known as oocyte spontaneous activation (4). Such spontaneous parthenogenetic activation may occur in human oocytes but mostly leads to tumor formation, including ovarian teratoma (OT) (5, 6). Some authors postulate that PG occurs in humans through the formation of OTs (2, 6, 7). However, they did not explain how this could happen. Although reproduction in most mammals occurs through mating between male and female, it has been hypothesized that presence of rare cases of PG in humans that result in normal and viable individuals go unnoticed due to the absence of congenital anomalies (8). Parthenogenetic activation by artificial manipulation has been conducted in mice with the creation of an early stage of fetal development (9). However, other authors stated that the creation and birth of the animal through oocyte parthenogenic activation using genetic modification technologies is possible (10).

This article aims to find the cause and mechanism of spontaneous PG and OT. Accordingly, we attempted to propose a new explanation for the occurrence of spontaneous PG in humans, which appears to occur more frequently than previously reported.

## 2. Materials and Methods

We searched the literature available in international journals and various scientific websites including Google Scholar, PubMed, Scopus, Web of Science, Springer, and Elsevier to find an explanation or pathogenesis for this phenomenon in humans. In this process, we used the title words as well as all subtitles and key words of the current article. For inclusion in this review, studies were case reports or original research dealing with spontaneous PG and its associated causes, risk factors, and proposed mechanism. Search limitations were to only include humans and English-language publications. We retrieved studies published up to December 2022. Then, we analyzed and discussed the results of previous relevant studies and added our proposal for an explanation for this rare event in humans.

## 3. Results and Discussion

PG or the so-called asexual reproduction that results in normal, viable offspring, is a rare event in humans (11). Previous literature on the causes of occurrence of these cases was sparse with no specific cause or mechanism. However, previous studies in their trial of finding an explanation for idiopathic OT, attributed these cases to spontaneous PG (2, 6, 7). This means that PG is not as rare as previously thought but can go unnoticed with an OT outcome or even a full-term birth, particularly, in the presence of a male partner.

### Specious variations in sexual and asexual reproduction

**Figure 2:**
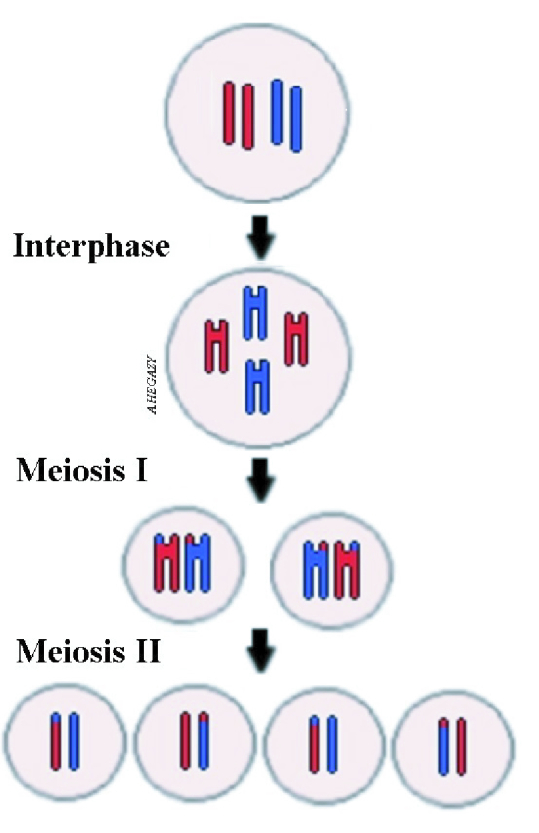
Diagrams showing exchange of chromosomes occurring during meiosis of gametogenesis.

Sexual reproduction occurs through the fertilization of a mature oocyte by a sperm, which leads to the restoration of the diploid number of the oocyte. Once the egg regains the 46 chromosomes, cell division by mitosis begins to form a new organism. In this type of reproduction, there will be diversity within the human species as well as sex determination. Species divergence occurs as half of the offspring's chromosomes come from the mother and the other from the father. On the other hand, sexual determination occurs through the sex chromosome carried by the sperm. In the case of X-chromosome sperm, the offspring will be female, while in the case of Y-chromosome sperm the creature will be male (3). This variation is not found in the case of PG where all chromosomes originate from the mother's side only; therefore, the new organism will be female “of the same sex as the mother.” In the process of PG, the female body finds a unique way to fill in the genes that sperm normally provide. However, the new organism may not typically be like its mother due to the exchange of chromosomes that occurs through the process of oogenesis (Figure 2).

### Reported cases of spontaneous PG

Spontaneous cases of PG have been reported in infertile women treated with assisted reproductive technologies (Table I) (6, 12-15). The diagnosis was made based on the findings that biopsy samples of embryos were exclusively compatible with those from the maternal side. It has been hypothesized that spontaneous stimulation of oocytes, or what is called PG, may be the factor involved in recurrent miscarriage and the inability to maintain a pregnancy (14). A case of recurrent miscarriage was reported with either in-vivo or in-vitro fertilization (IVF). Cytogenetic analysis of conceptus outcome samples, matched exclusively for the maternal side, may indicate that PG occurred spontaneously after insemination. On the other hand, other authors reported a case of PG embryo retrieved from an IVF patient with a history of infertility and OTs (6). They suggested presence of a possible link between spontaneous PG and OT development. Ye and co-authors also reported a case of PG in a 39-yr-old infertile woman (15). They supposed that occurrence of SG may be involved in failure to maintain pregnancy in IVF cases. Similarly, another case of a 38-yr-old infertile woman with a history of salpingectomy was also reported (14). It has been hypothesized that bilateral salpingectomy or advanced female age may result in spontaneous activation of oocytes (12, 14, 15). Other authors have suggested oocyte abnormalities in some infertile women that predispose to spontaneous PG (13, 14). Infertility was documented in all the cases mentioned. However, it is not clear whether exposure of the oocyte to sperm without fertilization or entry has a role in the spontaneous activation of the oocyte.

**Table 1:** Reported cases with PG in the previous literature


**Authors, Yr (Ref)**	**Country**	**Age (yr)**	**Gynecologic/Obstetric history**	**Outcome**
**Oliveira ** ***et al., *** **2004 (6) **	Brazil	29	Left oophorectomy due to ovarian teratoma	Pregnancy
**Combelles ** ***et al.,*** ** 2011 (13) **	USA	32	Recurrent miscarriages	No pregnancy
**Socolov ** ***et al.,*** ** 2015 (14) **	African ethnicity	38	Salpingectomy	No pregnancy
**Ye ** ***et al.,*** ** 2020 (15) **	China	38	Primary infertility with bilateral tubal obstruction	No pregnancy
**Jiang ** ***et al.,*** ** 2022 (12)**	China	33	Ectopic pregnancy; salpingectomy	Pregnancy

### Proposed automixis mechanism of PG

Fertilization in sexual reproduction occurs initially through the membrane of the SO that is normally prepared to penetrate through the rupture of the sperm cap, in order to release its enclosed enzymes such as acrosin- and trypsin-like substances that aid in sperm entry. The sperm capable of perforating the egg membrane is considered to be the healthiest to enable the new offspring to withstand the hardships of life in the future. After entering the fertilized sperm, the SO completes its second division to produce 2 cells: a large cell called the ovum whose nucleus fuses with the sperm nucleus to form the zygote, and the small SO which degenerates (Figure 1) (3).

**Figure 3:**
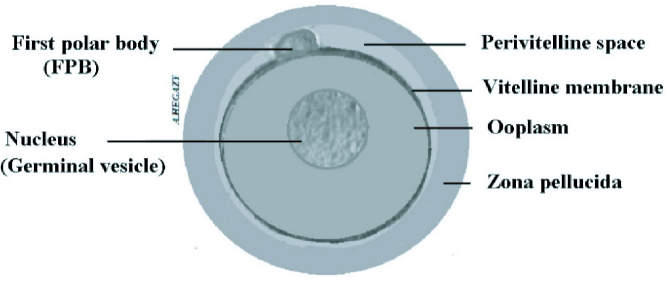
Diagram showing the 2 cells: SO and FPB surrounded by zona pellucida.

On the other hand, after the first meiosis of a primary oocyte with a diploid number “23 pairs” of chromosomes that occurs cyclically after female sexual puberty, 2 cells emerge, SO and first polar body (FPB). Each cell contains a haploid number of chromosomes “23." The process of release of SO from the surface of the ovary known as ovulation occurs once in each female reproductive cycle. The 2 cells “SO and FPB” are placed together within the zona pellucida (Figure 3).

Under normal conditions, FPB decomposes. However, we suggest that in some cases the 2 cells may fuse to form a single cell that restores the diploid number of chromosomes and initiates cell division to form a new organism. This may occur because of an aberration in the egg cell membrane, which may fuse with the membrane of FPB. The proposed automixis mechanism of PG was previously documented in animals such as sharks and aphids (16, 17). This condition is considered asexual reproduction but with no clonal offspring (17). This is because it depends on oogenesis, the process by which cross-over and exchange of DNA between chromosomes can occur during meiosis (Figure 2) (18).

**Figure 4:**
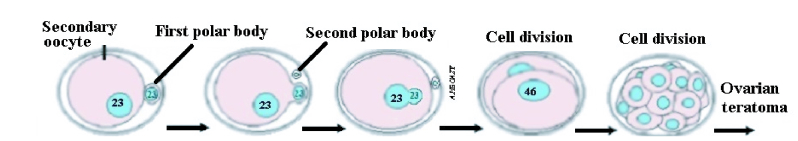
Diagrams showing the proposed automixis mechanism of PG and development of OT.

Therefore, PG differs from cloning in that the offspring is not as typical as its mother, while in cloning through the intracytoplasmic transfer of the somatic cell nucleus the new organism is typical with the donor (3). In this respect, the individual offspring of a typical parthenogenetic reproduction will be different from its mother and each other. Spontaneous PG in humans either degrades or leads to formation of OT (Figure 4), but it cannot develop into a term due to abnormal genomic imprinting and lack of paternal genes (4, 19). This is in addition to the aberrant location in the ovary. On the other hand, some authors have suggested another mechanism of PG. They attributed this to human embryonic stem cells of haploid oocytes that may have properties of pluripotent stem cells including self-renewal and a molecular signature of pluripotency (20).

### Teratoma formation theories

Teratomas are tumors made up of many tissues that differ in their location (21). Some theories have been stated to explain the cause of their appearance. The first theory supports evidence of origin from primordial germ cells. These cells arise from the embryonic endoderm of the yolk sac at the allantois and then migrate to the edge of the gonads. However, some of them miss their way and result in a teratoma anywhere from the brain to the sacrococcygeal region. Germ cell tumors are commonly encountered in fetuses and lead to neoplasms in the perinatal period. These tumors can be located in the gonads or outside along the midline (21, 22). The second theory attributes teratoma to incomplete twinning (22).

The third explanation stated that teratomas are remnants of the primitive embryonic node. This node gives rise to a primitive streak caudally that its cells migrate between the ectoderm and endoderm to form the third embryonic layer known as mesoderm. This last theory may explain the presence of teratoma in the sacrococcygeal region (21). However, all the previously mentioned theories miss the interpretation of the occurrence of teratoma in ovaries after puberty. Mature cystic teratoma is mostly found in females of reproductive age (23). Another theory claimed the existence of teratoma due to spontaneous oocyte activation but remains unclear. Recurrent spontaneous oocyte activation may be associated with infertility in women (24).

Despite the true causes for PG and OT are not clearly identified. There are some risk factors for formation of mature cystic OT. These include late menarche associated with menstrual irregularities, alcohol abuse, fewer pregnancies, and infertility (25). Other factors that can affect the ovarian reserve and that may cause aberration of ovarian cells include irradiation, malnutrition, drugs, and genetic factors (26).

### Triggers used for egg activation

The development of the embryo from the SO is achieved when the diploid number of chromosomes is restored. In sexual reproduction, fertilization itself activates the oocyte to form the new organism by increasing intracellular calcium within the SO (27). The calcium oscillations induced by the entry of sperm into the oocyte lead to oocyte activation to initiate embryo development. Oocyte activation events include cortical granules' exocytosis, recruitment of maternal mRNA, prevention of polyspermia, and pronuclear formation (28). This oocyte activation occurs in the case of cloning by somatic cell nucleus transfer through chemical stimulation or electrical impulses.

Moreover, activation of the oocyte could be induced in case of failure of intracytoplasmic sperm injection by calcium ionophores, including ionomycin or calcimycin. The calcium ionophores aid oocyte activation by increasing the permeability of the cell membrane, allowing extracellular calcium influx into the cell as well as aiding in the release of calcium from intracellular stores (29). Phospholipase C-zeta is one of the most important sperm-borne oocyte activation factors and represents a good biomarker in assessing the ability of oocytes to be activated (30, 31). We also suggest inducing such activation in the case of artificial PG.

Attempts to develop PG may outperform some other options, such as cloning via somatic cell nucleus transfer in producing stem cells or even creating a new organism after considering ethical issues. This is because the process of PG depends entirely on germ cells, while cloning depends on the transfer of nucleus of somatic cell of a donor into a previously enucleated oocyte. A somatic cell generally has a limited lifespan and the ability to divide to a limited extent and then go into senescence (32).

### Potential significance of the current review

It gives a new hypothesis for the rare phenomenon that may occur in all human generations. It may also explain some of the idiopathic tumors that occur in females such as the OT formed from different types of tissues, such as hair, muscle, bone, or teeth. A teratoma of the ovary is a benign tumor that can be converted into a malignant tumor. It consists of 3 embryonic layers called ectoderm, mesoderm, and endoderm (33). Teratomas represent the most common type of germ cell tumors. They occur mostly in the gonads. However, rare cases of extragonadal teratomas are documented, such as those in the body of the uterus (34).

Furthermore, PG could be developed to enhance the formation of stem cells and body tissues used in organ transplants and regenerative medicine in the future (1). Moreover, it can be an alternative to cloning in creating new organisms without sexual mating after taking ethical issues into account in humans.

## 4. Conclusion 

It is suggested that PG is a relatively common event and is not as rare in humans as we previously thought and can be represented in cases of the development of teratoma in the ovary. In this context, we propose the incorporation of SO and FPB as a possible explanation for PG and OT of reproductive age. FPB may also be injected into the SO, as in the case of intracytoplasmic sperm injection used in assisted reproductive techniques with ethical issues in mind. Finally, it remains a hypothesis until proven. It is recommended that the possibility of achieving this type of PG be investigated in future research to verify its feasibility.

### Limitations

The current work has some limitations, including the small number of studies included and some relatively old references. This may be due to a lack of proper attention in the previous literature, possibly due to the notion that it may be rare or even nonexistent in humans. Despite the apparent extreme rarity of its documented occurrence, the idea should not be ignored at all, especially if we agree to add the occurrence of teratoma of the ovaries as a type of PG. We hope that this work will draw the attention of scientists to study this phenomenon in humans, which does not appear to be as rare as previously thought.

##  Conflict of Interest

None.

## References

[bib1] Bos-Mikich A, Bressan FF, Ruggeri RR, Watanabe Y, Meirelles FV (2016). Parthenogenesis and human assisted reproduction. Stem Cells Int.

[bib2] de Carli GJ, Pereira TC (2017). On human parthenogenesis. Med Hypotheses.

[bib3] Hegazy A

[bib4] Cui W (2021). Oocyte spontaneous activation: An overlooked cellular event that impairs female fertility in mammals. Front Cell Dev Biol.

[bib5] Linder D, McCaw BK, Hecht F (1975). Parthenogenic origin of benign ovarian teratomas. N Engl J Med.

[bib6] Oliveira FG, Dozortsev D, Diamond MP, Fracasso A, Abdelmassih S, Abdelmassih V, et al (2004). Evidence of parthenogenetic origin of ovarian teratoma: Case report. Hum Reprod.

[bib7] Miura K, Kurabayashi T, Satoh C, Sasaki K, Ishiguro T, Yoshiura KI, et al (2017). Fetiform teratoma was a parthenogenetic tumor arising from a mature ovum. J Hum Genet.

[bib8] Strain L, Warner JP, Johnston T, Bonthron DT (1995). A human parthenogenetic chimaera. Nat Genet.

[bib9] Kaufman MH, Barton SC, Surani MAH (1977). Normal postimplantation development of mouse parthenogenetic embryos to the forelimb bud stag. Nature.

[bib10] Kono T, Obata Y, Wu Q, Niwa K, Ono Y, Yamamoto Y, et al (2004). Birth of parthenogenetic mice that can develop to adulthood. Nature.

[bib11] Paffoni A, Brevini TA, Somigliana E, Restelli L, Gandolfi F, Ragni G (2007). In vitro development of human oocytes after parthenogenetic activation or intracytoplasmic sperm injection. Fertil Steril.

[bib12] Jiang Y, Song G, Yuan JC, Zhang XH, Wu XH (2022). Genetic analysis of recurrent parthenogenesis: A case report and literature review. Exp Ther Med.

[bib13] Combelles CMH, Kearns WG, Fox JH, Racowsky C (2011). Cellular and genetic analysis of oocytes and embryos in a human case of spontaneous oocyte activation. Hum Reprod.

[bib14] Socolov R, Ebner T, Gorduza V, Martiniuc V, Angioni S, Socolov D (2015). Self-oocyte activation and parthenogenesis: An unusual outcome of a misconducted IVF cycle. Gynecol Endocrinol.

[bib15] Ye Y, Li N, Yan X, Wu R, Zhou W, Cheng L, et al (2020). Genetic analysis of embryo in a human case of spontaneous oocyte activation: A case report. Gynecol Endocrinol.

[bib16] Lampert KP

[bib17] Engelstädter J (2017). Asexual but not clonal: Evolutionary processes in automictic populations. Genetics.

[bib18] Pazhayam NM, Turcotte CA, Sekelsky J (2021). Meiotic crossover patterning. Front Cell Dev Biol.

[bib19] Li X, Zou C, Li M, Fang C, Li K, Liu Z, et al (2021). Transcriptome analysis of in vitro fertilization and parthenogenesis activation during early embryonic development in pigs. Genes (Basel).

[bib20] Sagi I, Chia G, Golan-Lev T, Peretz M, Weissbein U, Sui L, et al (2016). Derivation and differentiation of haploid human embryonic stem cells. Nature.

[bib21] Lakhoo K (2010). Neonatal teratomas. Early Hum Dev.

[bib22] Isaacs Jr H (2004). Perinatal (fetal and neonatal) germ cell tumors. J Pediatr Surg.

[bib23] Comerci Jr JT, Licciardi F, Bregh PA, Gregorgi C, Breen JL (1994). Mature cystic teratoma: A clinicopathologic evaluation of 517 cases and review of literature. Obstet Gynecol.

[bib24] Coskun S, Maddirevula S, Awartani K, Aldeery M, Qubbaj W, Kashir J, et al (2022). Recurrent spontaneous oocyte activation causes female infertility. J Assist Reprod Genet.

[bib25] Ahmed A, Lotfollahzadeh S https://www.ncbi.nlm.nih.gov/books/NBK564325/.

[bib26] Hegazy AA

[bib27] Horner VL, Wolfner MF (2008). Transitioning from egg to embryo: Triggers and mechanisms of egg activation. Dev Dyn.

[bib28] Nazarian H, Azad N, Nazari L, Piryaei A, Heidari MH, Masteri-Farahani R, et al (2019). Effect of artificial oocyte activation on intra-cytoplasmic sperm injection outcomes in patients with lower percentage of sperm containing phospholipase Cζ: A randomized clinical trial. J Reprod Infertil.

[bib29] Rahbaran M, Razeghian E, Maashi MS, Jalil AT, Widjaja G, Thangavelu L, et al (2021). Cloning and embryo splitting in mammalians: Brief history, methods, and achievements. Stem Cells Int.

[bib30] Meerschaut FV, Nikiforaki D, Heindryckx B, De Sutter P (2014). Assisted oocyte activation following ICSI fertilization failure. Reprod Biomed Online.

[bib31] Kashir J, Ganesh D, Jones C, Coward K (2022). Oocyte activation deficiency and assisted oocyte activation: Mechanisms, obstacles and prospects for clinical application. Hum Reprod Open.

[bib32] Mitalipov SM, Wolf DP (2000). Mammalian cloning: Possibilities and threats. Ann Med.

[bib33] Bonzi M, Fiorelli EM, Tobaldini E, Milani O, Bozzano V (2020). A giant ovary teratoma with malignant transformation. Intern Emerg Med.

[bib34] Zhao Y, Xu T, Bu X, Yuan D, Wu Y, Qian H (2021). Immature teratoma arising from uterine corpus in an 11-year-old girl: Case report and review of the literature. J Obstet Gynaecol Res.

